# Inflammation at diagnosis and cognitive impairment two years later in breast cancer patients from the Canto-Cog study

**DOI:** 10.1186/s13058-024-01850-5

**Published:** 2024-06-05

**Authors:** Mylène Duivon, Justine Lequesne, Antonio Di Meglio, Caroline Pradon, Ines Vaz-Luis, Anne-Laure Martin, Sibille Everhard, Sophie Broutin, Olivier Rigal, Chayma Bousrih, Christelle Lévy, Florence Lerebours, Marie Lange, Florence Joly

**Affiliations:** 1 ANTICIPE U1086 INSERM-UCN, Equipe Labellisée Ligue Contre Le Cancer, Centre François Baclesse, Normandie Université UNICAEN, 14000 Caen, France; 2 Clinical Research Department, UNICANCER, Centre François Baclesse, 3 Av. du Général Harris, 14000 Caen, France; 3 Services Unit PLATON, Cancer and Cognition Platform, University of Caen Normandy, 14000 Caen, France; 4 Cancer Survivorship Group, INSERM U981, Gustave Roussy, Villejuif, France; 5 Department of Medical Biology and Pathology, Gustave Roussy, Villejuif, France; 6 DIOPP, Gustave Roussy, Villejuif, France; 7 UNICANCER, 75654 Paris, France; 8 Biological Resource Center, AMMICa, INSERM US23/CNRS UMS3655, Gustave Roussy, Villejuif, France; 9 Care Support Department, Centre Henri Becquerel, 76000 Rouen, France; 10 Medical Oncology Department, Centre Henri Becquerel, 76000 Rouen, France; 11 Gustave Roussy, 94800 Villejuif, France; 12 Institut Normand du Sein, Centre François Baclesse, 14000 Caen, France; 13 Medical Oncology Department, Institut Curie, 92210 Saint Cloud, France

**Keywords:** Breast cancer, Cytokines, Cancer-related cognitive impairment, C-reactive protein

## Abstract

**Background:**

Inflammation could be related to cancer-related cognitive impairment (CRCI) and might be used as a predictive marker of long-term CRCI. We evaluated associations between inflammatory markers assessed at diagnosis of breast cancer and CRCI two years afterwards.

**Methods:**

Newly diagnosed stage I-III patients with breast cancer from the French CANTO-Cog (Cognitive sub-study of CANTO, NCT01993498) were included at diagnosis (baseline). Serum inflammatory markers (IL-2, IL-4, IL-6, IL-8, IL-10, TNFα, CRP) were assessed at baseline. Outcomes at year 2 post-baseline included overall cognitive impairment (≥ 2 impaired domains) and the following domains: episodic memory, working memory, attention, processing speed, and executive functions. Multivariable logistic regression models evaluated associations between markers and outcomes, controlling for age, education, and baseline cognitive impairment.

**Results:**

Among 200 patients, the mean age was 54 ± 11 years, with 127 (64%) receiving chemotherapy. Fifty-three (27%) patients had overall cognitive impairment at both timepoints. Overall cognitive impairment at year 2 was associated with high (> 3 mg/L) baseline CRP (OR = 2.84, 95%CI: 1.06–7.64, *p* = 0.037). In addition, associations were found between high CRP and processing speed impairment (OR = 2.47, 95%CI:1.05–5.87, *p* = 0.039), and between high IL-6 and episodic memory impairment (OR = 5.50, 95%CI:1.43–36.6, *p* = 0.010).

**Conclusions:**

In this cohort, high levels of CRP and IL-6 assessed at diagnosis were associated with overall CRCI, processing speed and episodic memory impairments two years later. These findings suggest a potential inflammatory basis for long-term CRCI. CRP may represent an easily measurable marker in clinical settings and be potentially used to screen patients at greater risk of persistent CRCI.

**Supplementary Information:**

The online version contains supplementary material available at 10.1186/s13058-024-01850-5.

## Background

Cancer-related cognitive impairment (CRCI) is a symptom that frequently affects the quality of life of patients with breast cancer [1]. CRCI refers to complaints about difficulty in remembering things and concentrating, the most affected domains usually being memory, attention, executive functions and processing speed [2, 3]. These difficulties may be observed before any treatment [4] and may last for years after cancer diagnosis [5]. We previously found that around 30% of survivors of breast cancer had CRCI before treatment and two years after cancer diagnosis [6]. Regarding the etiology of CRCI, numerous risk factors have been identified such as aging, chemotherapy, psychological factors and baseline cognition. Nevertheless, the potential biological mechanisms underlying CRCI and associated factors require further investigation.

A focus to date has been to identify easily measurable biological markers in breast cancer patients, particularly serum inflammation. Previous studies showed an association between inflammatory markers (e.g. cytokines, C-reactive protein, lymphocytes) and cognitive performances, both mostly studied after the end of cancer treatments [7–11]. This association can in part be explained by inflammation-related signaling and damage in the brain. Inflammation, combined with drug cytotoxicity—particularly from chemotherapy—may change the structure and integrity of the blood brain barrier. This may allow cytokines to pass through and impact cortical integrity, thereby contributing to CRCI [12]. Nevertheless, there is a lack of consensus due to methodological issues like the choice of variables of interest (biological markers, cognitive evaluation, factors of adjustment), the measurement and analysis used, and the timing of assessment [9, 13–15]. Two recent studies found that inflammation before chemotherapy was associated with cognitive complaints one year later and was related with a decline in attention and processing speed performances after chemotherapy [16, 17]. To our knowledge, no study until now has evaluated the association between inflammatory markers measured before any treatment, including surgery, and objective CRCI post-treatment. In this study, we investigated the cognitive outcomes of breast cancer patients included in the CANTO-Cog sub-study of the French CANTO cohort. We examined pre-treatment inflammatory marker levels and their association with CRCI two years after diagnosis. We hypothesized that higher levels of pro-inflammatory markers would be associated with an overall cognitive impairment two years later. Exploratory analyses were performed to assess the association between each inflammatory marker and the cognitive domains measured.

## Methods

### Patients

The data used to conduct this study were obtained from a cognitive sub-study (CANTO-Cog) of the French CANTO cohort (CANcer TOxicities, ClinicalTrials.gov identifier: NCT01993498), which included women newly diagnosed with localized, stage I-III breast cancer [18]. This sub-study, which was previously described [4], concerns the longitudinal investigation of cognitive functioning. Eligible patients had not yet received cancer treatment (including surgical treatment for current breast cancer), were conversant in the French language, had no neurological or psychiatric comorbidities, no alcohol or drug abuse, a formal education ≥ 5 years (end of primary school) and no major cognitive disorders (i.e., Mini-Mental State Examination [19] score ≥ 26 out of 30).

All participants in the CANTO-Cog sub-study provided written informed consent and the study was approved by the ethics committee (ID-RCB:2011-A01095-36,11–039). Data were collected at diagnosis, i.e., before any cancer treatment (baseline), and two years after diagnosis (year 2) corresponding roughly to one year after primary treatment completion (surgery, chemotherapy, and/or radiation therapy).

### Variables of interest

#### Cognitive outcomes

Objective cognitive functioning was measured with nine paper-based standardized neuropsychological tests: Hopkins Verbal Learning Test Revised [20], d2 [21], Verbal fluency [22], Trail Making Test [23], Stroop test [24], Spatial span [25], Digit span [25], Letter Number Sequencing [25], and Symbol search [25]. As previously described in Lange et al., 2020, test scores were corrected for practice effect and standardized as z-scores using the means and standard deviations of the healthy control group recruited for the CANTO-Cog study [4]. Neuropsychological z-scores were averaged to create a composite z-score for each of the five cognitive domains (described elsewhere [4]): episodic memory, working memory, information processing speed, attention, and executive function.

A cognitive domain was considered as impaired according to International Cognition and Cancer Task Force (ICCTF) recommendations if at least two test scores were below –1.5 standard deviations or one single test score was below − 2.0 standard deviations from the mean of the healthy control group [26].

The primary outcome of interest was the overall cognitive impairment, defined as having at least two impaired cognitive domains. The secondary outcome was cognitive impairment in each cognitive domain.

#### Covariates

Covariates included demographic (age and level of education), clinical (body mass index, BMI) and treatment (chemotherapy) characteristics. Anxiety and depression were measured with the Hospital Anxiety Depression Scale (HADS) [27], with a score ≥ 11 considered as severe symptoms. Physical, emotional and cognitive fatigue were measured with the EORTC QLQ-FA12 scale [28], with a score ≥ 40 considered as severe fatigue. Baseline cognitive impairment was measured as previously described.

#### Marker variables

Inflammation markers were measured with blood samples profiled at baseline using high sensitivity multiplex protein arrays provided by RANDOX Laboratories Limited (Crumlin, United Kingdom): Cytokine Custom array (CTK CST X, EV3881/EV3623), Metabolic Syndrome array I (METS I, EV3755) and Metabolic Syndrome array II (METS II, EV3759/A). The RANDOX Evidence Investigator™ Biochip Array technology was used, enabling simultaneous quantitative detection of multiple analytes from a single sample. Analytical procedures were followed according to the manufacturer's instructions. Using the available literature on cognition and inflammation, we chose markers demonstrating consistent associations with CRCI: IL-2, IL-4, IL-8, IL-10 (CTK), IL-6, TNFα (METSI), CRP (METSII) [13, 14, 29–37].

Data on inflammatory markers were analyzed as categorical variables based on the lower limit of quantification for the individual assay (functional sensitivity threshold). The sensitivity of the Evidence Investigator™ assays was determined as the minimum level at which the imprecision did not exceed 20% across 20 replicates. If more than 80% of inflammatory marker levels were below the sensitivity threshold of the Randox array, the marker was removed from the analyses. If fewer than 25% of inflammatory marker levels were below the sensitivity threshold of the Randox array, the marker values were analyzed as continuous. Otherwise, i.e. between 25 and 80% of marker levels were below the sensitivity threshold, the marker values were dichotomized as “low” vs “high” according to whether they were below or above the threshold, respectively [38]. CRP levels were categorized according to the clinical threshold of 3 mg/L found in the literature [17] and Randox array norms, e.g. CRP > 3 mg/L is considered as clinically high inflammation.

### Statistical analysis

Descriptive statistics were used to summarize the socio-demographic, clinical and biological variables of all patients at baseline and according to overall cognitive impairment at year 2. Comparisons were conducted using Chi-square, Student, or Wilcoxon tests, as appropriate for categorical and continuous variables.

In our initial approach, we built distinct logistic regression models for overall cognitive impairment and each cognitive domain. Each model was adjusted for age (continuous), years of education (continuous) and cognitive impairment at baseline (yes/no). We investigated the association between inflammation and cognition, building a model for each marker. At the same time, we explored various adjustment factors and built a model for each of the following: anxiety at year 2 (yes/no), depression at year 2 (yes/no), BMI at baseline (continuous scores), cognitive/emotional/physical fatigue at year 2 (continuous scores), and chemotherapy treatment (yes/no).

Next, inflammatory markers identified in previous logistic regression models as significantly associated with cognitive impairment were incorporated into a single multivariable logistic regression model. This model was adjusted for cognitive impairment at baseline, age, and level of education, along with adjustment factors that were significantly related with cognitive impairment in models of the initial approach. This comprehensive model was created for overall cognitive impairment and each cognitive domain if multiple inflammatory markers were found to be associated with the same cognitive outcome. Ultimately, a backward elimination procedure based on significance (*p* < 0.05) was implemented to derive the best fit model.

All statistical analyses were carried out with R software. A *p* value < 0.05 was considered statistically significant in each analysis.

## Results

**Fig. 1:**
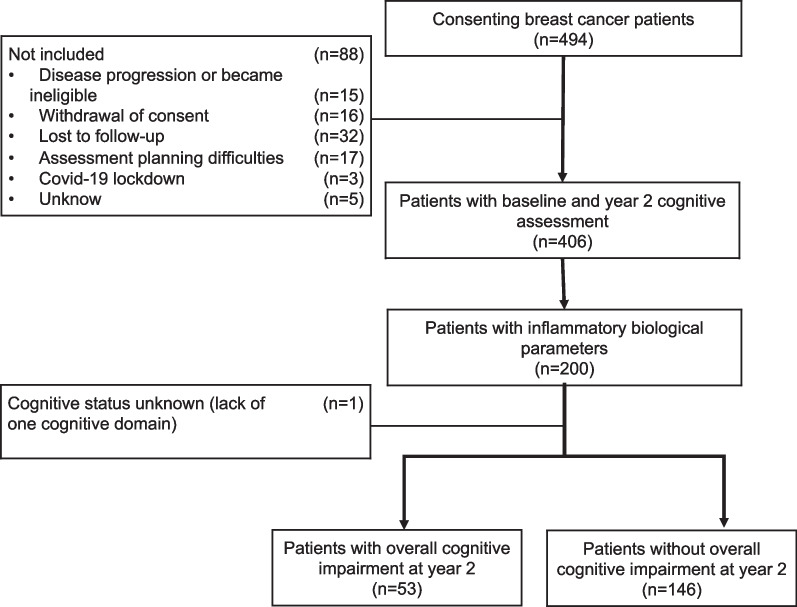
Flow chart of CANTO-Cog study

Among the 494 patients who consented to participate in the CANTO-Cog sub-study, 200 patients were included in the analysis (Fig. 1).

### Patient characteristics, patient-reported outcomes, and cognitive impairment

Patient characteristics are shown in Table 1. Mean age was 54 ± 11 years at baseline, 168 patients (85%) had a stage I or II breast cancer, 127 patients (64%) were treated with chemotherapy, 190 (95%) with radiotherapy and 164 (82%) with endocrine therapy. At year 2, 37 (18%) patients had clinical symptoms of anxiety, 13 (6%) depression, 24 (12%) cognitive fatigue, 37 (18%) emotional fatigue, and 62 (31%) physical fatigue (mean scores in Additional file 1).

**Table 1:** Characteristics of all patients, and according to overall cognitive impairment status at year 2

Characteristics	Patients (n = 200)	Patients with overall impairment at year 2 (n = 53)	Patients without overall impairment at year 2 (n = 146)	*p*
**Demographics**				
Age, mean years (SD) [range]	53.5 (11) [28–83]	58.1 (10) [35–81]	51.9 (11) [28–83]	< 0.001
Education, mean years (SD) [range]	13.2 (2.8) [5–20]	11.7 (2.7) [5–20]	13.8 (2.6) [9–20]	< 0.001
**Clinical**				
ECOG, No. (%)				1
0	185 (97)	49 (98)	136 (97)	
1 ≤	5 (3)	1 (2)	4 (3)	
Missing	10 (5)	2 (3.8)	8 (5.6)	
Charlson, No. (%)				0.05
0	148 (78)	33 (67)	114 (82)	
1 ≤	41 (22)	16 (33)	25 (18)	
Missing	11 (5.5)	4 (7.5)	7 (4.9)	
Psychotropic medications, No. (%)	15 (8)	5 (9)	10 (7)	0.76
BMI, mean (SD)	26.0 (5.2)	27.5 (5.8)	25.5 (4.9)	0.03
Cancer stage, No. (%)				0.07
Stage I	85 (43)	27 (51)	57 (39)	
Stage II	83 (42)	17 (32)	66 (46)	
Stage III	30 (15)	9 (14)	21 (15)	
Missing	2 (1)	0 (0)	2 (1.4)	
Grade, No. (%)				0.35
I	29 (15)	10 (20)	19 (13)	
II	105 (53)	28 (55)	76 (52)	
III	63 (32)	13 (25)	50 (34)	
Missing	3 (1.5)	1 (1.9)	2 (1.4)	
HER2-Positive, No. (%)	25 (13)	7 (13)	18 (12)	1
Negative	174 (87)	45 (87)	128 (88)	
Missing	1 (0.5)	1 (1.9)	0 (0)	

*BMI* Body mass index; *ECOG* Eastern cooperative oncology group

Fifty-three patients had overall cognitive impairment at both time points (27%). Patients with overall cognitive impairment at year 2 were older (*p* < 0.001), had a lower level of education (*p* < 0.001), and higher BMI (*p* = 0.03) at baseline than patients without impairment. At year 2, patients with overall cognitive impairment had a higher HADS depression score (*p* = 0.03) than patients without overall cognitive impairment at year 2 (Additional file 1).

Z-scores in each cognitive domain are reported in Table 2. Processing speed was the most affected cognitive domain as 33% of patients demonstrated impairment at both time points.

**Table 2:** Cognitive assessment at baseline and year 2

Cognitive domains	Baseline (n = 200)		Year 2 (n = 200)	
	Z-score, mean(SD)	No. (% impaired)	Z-score, mean(SD)	No. (% impaired)
Overall	NA	53 (27)	NA	53 (27)
Episodic Memory	− 0.11 (0.81)	35 (18)	− 0.02 (0.85)	26 (13)
Working Memory	− 0.46 (0.72)	34 (17)	− 0.33 (0.85)	35 (18)
Processing Speed	− 0.34 (0.74)	66 (33)	− 0.33 (0.85)	65 (33)
Attention	− 0.33 (0.92)	41 (21)	− 0.20 (0.96)	37 (19)
Executive Function	− 0.09 (0.61)	37 (19)	− 0.11 (0.57)	44 (22)

### Inflammatory markers

After checking the marker levels based on the functional sensitivity threshold (Additional file 2), we finally analyzed the following cytokines: IL-6 and CRP (as categorial variables), IL-8 and TNFα (analyzed as continuous variables).

**Fig. 2:**
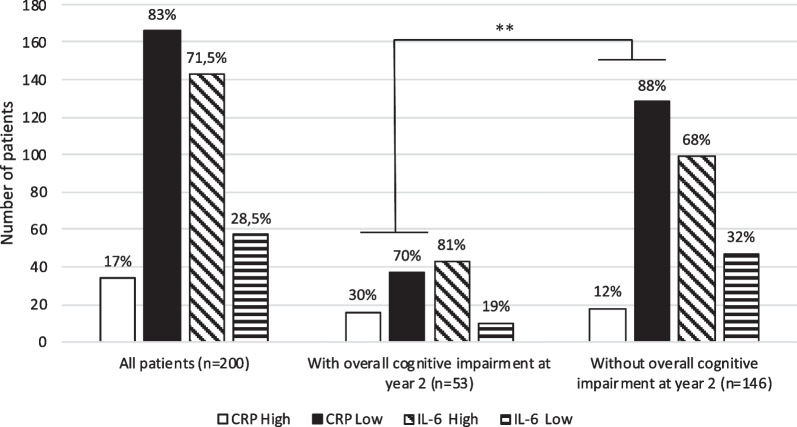
Number (and percentage) of patients with high vs. low CRP and IL-6 levels. CRP: C-reactive protein; IL-6: Interleukin 6; High: level above threshold; Low: level below threshold; ** *p* value < 0.01

Thirty-four (17%) patients at baseline had CRP > 3 mg/L (Fig. 2). Patients with overall cognitive impairment at year 2 were more likely to have a high CRP level at baseline (n = 16, 30%) than patients without it (n = 18, 12%; *p* = 0.006). Patients with overall cognitive impairment at year 2 tended to be more likely to have a high IL-6 level (n = 43, 81%) than patients without it (n = 99, 68%; *p* = 0.10). No significant between-group difference was observed for IL-8 and TNFα levels (Additional file 2).

### Association between overall cognitive impairment at year 2 and inflammatory markers at baseline

Models of the initial approach demonstrated an association between overall cognitive impairment at year 2 and baseline levels of IL-8 (OR = 0.85, 95%CI: 0.72–0.98, *p* = 0.02), as well as CRP (OR = 2.8, 95%IC: 1.10–7.66, *p* = 0.03). This suggests that lower IL-8 levels and high CRP levels were associated with overall cognitive impairment at year 2 (Table 3). No adjustment factors were associated with overall cognitive impairment, so the unique model was adjusted on age, education, and cognition at baseline. When included in a unique model, only the association with CRP remained significant (OR = 2.84, 95%CI: 1.06–7.64, *p* = 0.04).

**Table 3:** Association between level of inflammatory markers at baseline and overall cognitive impairment at year 2 (n = 196)

Inflammatory markers at baseline	Overall cognitive impairment at year 2			
	Models (for each marker)		Unique model	
	OR (95% CI)	*p*	OR (95% CI)	*p*
IL-6	1.48 (0.59 to 3.88)	0.41	–	–
IL-8	0.85 (0.72 to 0.98)	0.02	0.85 (0.71 to 0.98)	0.06
CRP > 3	2.89 (1.10 to 7.66)	0.03	2.84 (1.06 to 7.64)	0.04
TNFα	0.93 (0.70 to 1.19)	0.57	–	–

Models and unique model are adjusted on age, years of education, and cognitive impairment at inclusion. Unique model includes inflammatory markers related with overall cognitive impairment (*p* < 0.05) in previous models, followed by backward elimination (*p* < 0.05)

### Association between impaired cognitive domain at year 2 and inflammatory markers at baseline

High IL-6 and CRP levels at baseline were associated with impairment in episodic memory (OR = 5.50, 95%CI: 1.43–36.6, *p* = 0.03) and processing speed (OR = 2.47, 95%CI: 1.05–5.87, *p* = 0.04) at year 2, respectively (Table 4). Lower baseline TNFα levels were associated with working memory (OR = 0.64, 95%CI: 0.44–0.89, *p* = 0.01) impairment at year 2. No adjustment factors were related with impaired cognitive domains, and there was no instance of multiple inflammatory markers being associated with the same cognitive domain. Consequently, there was no need for a unique model.

**Table 4:** Association between inflammation at baseline and cognitive domains impaired at year 2

Inflammatory markers at baseline	Episodic memory (n = 200)		Working memory (n = 197)		Processing speed (n = 197)		Attention (n = 194)		Executive function (n = 196)	
	OR (95% CI)	*P*	OR (95% CI)	*P*	OR (95% CI)	*P*	OR (95% CI)	*P*	OR (95% CI)	*P*
IL-6	5.50 (1.43 to 36.60)	0.03	0.98 (0.37 to 2.79)	0.97	1.47(0.68 to 3.30)	0.33	0.79 (0.24 to 2.58)	0.69	0.75 (0.34 to 1.73)	0.49
IL-8	0.94 (0.75 to 1.06)	0.51	0.93 (0.76 to 1.05)	0.42	0.96(0.85 to 1.04)	0.40	0.91 (0.79 to 1.03)	0.17	0.85 (0.69 to 0.98)	0.07
CRP > 3	2.59 (0.91 to 7.04)	0.07	1.23 (0.41 to 3.36)	0.70	2.47(1.05 to 5.87)	0.04	1.01 (0.24 to 4.04)	0.99	2.04 (0.81 to 4.92)	0.12
TNFα	1.25 (0.95 to 1.63)	0.09	0.64 (0.44 to 0.89)	0.01	1.03(0.83 to 1.26)	0.82	0.8 (0.53 to 1.16)	0.26	0.93 (0.72 to 1.19)	0.59

Different model for each marker, adjusted on age, years of education, cognitive impairment at inclusion

## Discussion

To our knowledge, this is the first study conducted on a national cohort to evaluate the association between inflammatory markers measured at diagnosis and CRCI two years later. CRP > 3 mg/L at diagnosis of an invasive localized breast cancer was associated with overall cognitive impairment at year 2. Moreover, high IL-6 and CRP levels at diagnosis were associated with impaired episodic memory and processing speed, respectively. We suggest that serum inflammatory markers at diagnosis could contribute to identifying patients at a higher risk of long-term CRCI after treatment.

Patients with overall cognitive impairment at year 2 had higher CRP levels at baseline than those without it. CRP, which is a sensitive indicator of inflammation that is easily measurable in clinical settings, has already been associated with cognitive impairment and was shown to be a predictive marker of dementia in healthy older adults [39–41]. Carroll et al. also recently suggested that it could be used to screen patients over 60 years of age with cognitive complaints after treatment [17]. In that study, breast cancer survivors were evaluated every year from pre-systemic therapy to 60 months post-therapy: CRP evaluated at one study visit was related with cognitive complaints (no significant relationship with objective cognitive impairment) during the subsequent visit, i.e., one year later. Our study included patients aged at least 18 years with a median of age of 53 years, so CRP could also serve to screen middle-aged patients at greater risk of CRCI after treatment.

Another aim was to explore the association between inflammatory markers and various domains of cognition. We found an association between high CRP levels and processing speed impairment, which was the most impaired domain in our population. Starkweather et al. also found an association between higher cognitive functioning, including processing speed, and lower CRP levels during and after chemotherapy [42]. More recently, Belcher et al. evaluated the association between inflammation markers (CRP not measured) before chemotherapy and processing speed/attention decline after treatment. They identified an association between inflammation (lower level of IL-4 and higher level of TNFRI-II) before chemotherapy, and greater processing speed/attention decline after chemotherapy [16]. They suggested that inflammatory markers may improve the precision of predicting the risk of developing CRCI. Although the markers used in our study differ, we also observed that a high level of inflammation before treatment, even surgery, is associated with processing speed impairment after treatment.

In the current study, high IL-6 levels at diagnosis were associated with episodic memory impairment two years later. The role of IL-6 in episodic memory functioning has been demonstrated in studies on mice [43, 44] and patients with Alzheimer’s disease [45, 46]. An association between IL-6 and memory has also been observed in cross-sectional studies in patients with breast cancer [30, 35]. Shibayama et al. found that IL-6 mediated episodic memory performances in patients treated with radiotherapy, and Kesler et al. found that breast cancer patients had higher IL-6 levels, reduced hippocampal volume and memory performances than healthy controls [30, 35]. Our patients with CRCI at year 2 tended to have higher baseline IL-6 levels than those without it. This lack of significance could be explained by the threshold used, which unlike CRP is not a validated clinical threshold. IL-6 has recently been found to mediate the relationship between survival after breast cancer and CRCI [10]. Mandelblatt et al. suggested that this strong association between IL-6 and cognition may be partly due to the fact that a low level of IL-6 is needed to trigger an inflammatory response. Thus, even with a low level of inflammation, IL-6 could serve as a predictive marker. Nevertheless, a clinical threshold is required for routine clinical use. Altogether, previous studies and the present findings indicate that IL-6 may play a role in long-term CRCI and particularly on episodic memory impairment after cancer treatment, and that it might be used as a predictive marker.

Unexpectedly, we found that lower TNFα levels at diagnosis were associated with working memory impairment two years later. In the literature, higher TNFα levels in patients with breast cancer have been associated with greater objective and subjective cognitive impairment [14, 30, 31, 37, 47]. Toh et al. found associations between cytokine fluctuations and cognitive trajectories, but the association was significant for TNFα only weeks after chemotherapy initiation [14]. The authors hypothesized that TNFα might be involved in the acute phase of immune and CRCI response, whereas other cytokines such as IL-6 and IL-8 related with persistent CRCI might be affected rather by the inflammatory environment and could play a role in regulating the autoimmunity/chronic inflammatory disease [14]. Williams et al. also found that TNFα was no longer significantly associated with processing speed performance when anthracycline exposure was added to the model [48]. Taken together, results from previous studies suggest that TNFα is strongly related to response to chemotherapy. In our study, where 64% of patients underwent chemotherapy, cognition was evaluated more than one year after chemotherapy treatment, i.e., after the acute effect of chemotherapy on cognition (no association observed between previous chemotherapy treatment and cognitive impairment at year 2, results not shown). Hence, one could hypothesize that while TNFα may not be effective in predicting long-term CRCI, it might be more suitable for understanding the biological mechanisms of CRCI during and shortly after chemotherapy.

CRCI has multifactorial causes including depression, anxiety, and fatigue, which are also known to be associated with inflammation [42, 49–51]. A recent study found that higher psychological distress indirectly predicted worse cognition through higher levels of IL-1β, TNF-α, and IL-4 [52]. In our study, we did not find a significant association between objective cognitive impairment and depression, anxiety, fatigue and, BMI (results not shown).

This study has some limitations. First, a high percentage of marker levels fell below the functional sensitivity threshold, so we analyzed continuous data as categorical data. This categorization was necessary for our CANTO cohort but is not generalizable to all cancer populations. Second, the mean level of each inflammation marker was within the normal range of the Randox arrays or those provided by the Mayo Clinic laboratories website [53], which could have limited the strength and number of associations found. Finally, we could not use inflammatory data from our control cohort (included in CANTO-Cog) for the analyses since the healthy controls did not have blood tests.

The strengths of this study lie in the use of a large national cohort, the acquisition of baseline data before any treatment, the use of validated neuropsychological tests in accordance with ICCTF recommendations performed prior to any treatment, including surgery, and its longitudinal design. Furthermore, the practice effect was controlled for analysis, and statistical models were adjusted for cognitive performances at baseline, i.e. time of measuring inflammatory markers.

## Conclusions

Our findings could have a direct impact by allowing clinicians to better evaluate pre-treatment factors related with CRCI over time. A CRP assay could be added systematically to routine blood testing before surgery for the early identification of patients at greater risk of long-term CRCI. Although not yet suitable for clinical use, IL-6 could be used to detect a greater risk of episodic memory impairment, whereas the use of TNFα as a predictor of CRCI remains to be confirmed. As a preventive measure, patients with a high level of inflammation (e.g. CRP > 3 mg/L) could be offered interventions such as physical activity, which is known to improve inflammation [54–56] and cognition [57, 58], psycho-education and/or cognitive training sessions [59].

### Supplementary Information

**Additional file 1.** Clinical characteristics and patients reported outcomes at year-2.

**Additional file 2.** Inflammatory characteristics of patients at baseline.

## Data Availability

The datasets used and/or analysed during the current study are available from c-gaudin@unicancer.fr on reasonable request.

## Abbreviations

BMI: Body mass index
CANTO: CANcer TOxicities
CRCI: Cancer-related cognitive impairment
CRP: C-reactive protein
EORTC QLQ-FA12: European organization for research and treatment of cancer quality of life questionnaire core 30—fatigue module 12 items
ICCTF: International cancer and cognition task force
HADS: Hospital anxiety depression score
IL: Interleukin
TNF: Tumor necrosis factor
