# Supplementary material for: Inflammation at diagnosis and cognitive impairment two years later in breast cancer patients from the Canto-Cog study
Source: Breast Cancer Res. 2024 Jun 5;26:93. doi: 10.1186/s13058-024-01850-5 (PMC11151649; doi:10.1186/s13058-024-01850-5)
Supplement: Supplementary file 1 — Additional file 1. Clinical characteristics and patients reported outcomes at year-2. [file 13058_2024_1850_MOESM1_ESM.docx]

Additional file 1. Clinical characteristics and patients reported outcomes at year-2

| **Characteristics** | **All patients (n=200)** | **Patients with overall cognitive impairment at year-2 (n=53)** | **Patients without overall cognitive impairment at year-2 (n=146)** | **P** |
| --- | --- | --- | --- | --- |
| **Type of invasive cancer, No. (%)** | | | | .99 |
| Ductal | 161 (80) | 43 (81) | 117 (80) |  |
| Lobular | 23 (12) | 6 (11) | 17 (11) |  |
| Mixed | 16 (8) | 4 (8) | 12 (8) |  |
| **Treatments, No. (%)** | | | | |
| Lumpectomy | 157 (78) | 114 (78) | 42 (79) | 1 |
| Mastectomy | 49 (24) | 36 (25) | 13 (25) | 1 |
| Sentinel node biopsy | 151 (76) | 39 (74) | 111 (76) | .87 |
| Axillary clearance | 79 (40) | 20 (38) | 59 (40) | .86 |
| Chemotherapy | 127 (64) | 95 (65) | 32 (60) | .66 |
| Radiation therapy | 190 (95) | 141 (97) | 48 (91) | .18 |
| Endocrine therapy | 164 (82) | 122 (84) | 41 (77) | .43 |
| Herceptin | 23 (12) | 7 (13) | 16 (11) | .85 |
| **Patients reported outcomes, mean (SD)** | | | | |
| Anxiety | 6.82 (4.0) | 6.77 (4.3) | 6.84 (3.9) | .92 |
| Depression | 4.01 (3.6) | 5.08 (4.5) | 3.61 (3.1) | .03 |
| Physical fatigue | 31.8 (24) | 3.4 (23) | 32.4 (24) | .61 |
| Emotional fatigue | 2.4 (27) | 18.9 (26) | 2.9 (27) | .63 |
| Cognitive fatigue | 17.8 (24) | 17.0 (24) | 18.2 (24) | .76 |
