# Supplementary material for: Inflammation at diagnosis and cognitive impairment two years later in breast cancer patients from the Canto-Cog study
Source: Breast Cancer Res. 2024 Jun 5;26:93. doi: 10.1186/s13058-024-01850-5 (PMC11151649; doi:10.1186/s13058-024-01850-5)
Supplement: Supplementary file 2 — Additional file 2. Inflammatory characteristics of patients at baseline. [file 13058_2024_1850_MOESM2_ESM.docx]

Additional file 2. Inflammatory characteristics of patients at baseline

| **Markers (sensitivity threshold)** | **Patients with concentration under threshold**  **(n=200)**  **No. (%)** | **Marker concentration of all patients (n=200)**  **Median [IQR]** | **Patients with overall cognitive impairment at year-2 (n=53)**  **Median [IQR]** | **Patients without overall cognitive impairment at year-2 (n=144)**  **Median [IQR]** | **P** |
| --- | --- | --- | --- | --- | --- |
| CRP (>3mg/L) | 50 (25) | 1.12 [0.70-2.13] | 1.51 [0.87-3.25] | 1.01 [0.64-1.81] | .004 |
| IL-2 (2.97pg/ml) | 82 (90) | 1.12 [0.93-1.49] | 1.21 [0.915-1.44] | 1.11 [0.940-1.56] | .96 |
| Missing No. (%) |  | 17 (8.5) | NA | NA |  |
| IL-4 (2.12pg/ml) | 192 (96) | 0.77 [0.64-0.96] | 0.74 [0.71-0.93] | 0.78 [0.63-0.96] | .79 |
| Missing No. (%) |  | 1 (0.5) | NA | NA |  |
| IL-6 (0.73pg/ml) | 57 (29) | 0.97 [0.67-1.57] | 1.17 [0.79-2.12] | 0.93 [0.66-1.45] | .05 |
| IL-8 (0.36pg/ml) | 1 (0.5) | 3.12 [2.20-4.64] | 3.75 [2.37-5.22] | 2.98 [2.19-4.53] | .09 |
| Missing No. (%) |  | 1 (0.5) | 0 | 0 (0.7) |  |
| IL-10 (0.37pg/ml) | 163 (81) | 4.72 [4.02-5.84] | 4.7 [4.16-5.86] | 4.75 [3.88-5.82] | .52 |
| Missing No. (%) |  | 3 (1.5) | NA | NA |  |
| TNFα (0.66pg/ml) | 0 (0) | 1.35 [1.11-1.58] | 1.37 [1.08-1.59] | 1.29 [1.11-1.56] | .68 |

Number of patients under the functional sensitivity threshold and concentration of inflammatory markers at baseline
